# RING Finger Protein 10 Regulates AP-1/Meox2 to Mediate Pirarubicin-Induced Cardiomyocyte Apoptosis

**DOI:** 10.1155/2023/7872193

**Published:** 2023-01-20

**Authors:** Hongwei Shi, Liang Duan, Ying Lan, Quan He, Peng Pu, Heng Tang

**Affiliations:** ^1^Department of Radiation Oncology, Hubei Cancer Hospital, Tongji Medical College, Huazhong University of Science and Technology, Wuhan, China; ^2^Department of Oncology, Renmin Hospital of Wuhan University, Wuhan, China; ^3^Department of Cardiology, The First Affiliated Hospital of Chongqing Medical University, Chongqing, China; ^4^Department of Cardiology, Southwest Hospital, Third Military Medical University (Army Medical University), Chongqing 400038, China

## Abstract

Pirarubicin (THP) is one of the classic chemotherapy drugs for cancer treatment. It is often clinically limited because of its cardiotoxicity. The occurrence and development of THP-mediated chemotherapy-related cardiotoxicity (CRC) may be reversed by RING finger protein 10 (RNF10). This study was performed with the aim of evaluating the inhibitory effect of RNF10 on THP-mediated CRC and its molecular mechanism. In vivo, we found that the expression of RNF10 decreased in THP-induced CRC rats, accompanied by Meox2 inhibition and AP-1 activation, resulting in increased cardiomyocyte apoptosis. After small interfering RNA (siRNA) and lentivirus transfection (Lv) of RNF10 in vitro, the expression of RNF10, Meox2, and AP-1 proteins and the degree of cardiomyocyte apoptosis were detected. We found that overexpression of RNF10 in H9C2 cardiomyocytes significantly promoted Meox2 and inhibited AP-1, alleviated apoptosis, and showed further inhibitory activity on THP-induced cardiomyocyte toxicity. Silencing RNF10 showed the opposite result. Our study showed that RNF10 inhibited THP-induced CRC through the activity of Meox2 and AP-1 proteins. RNF10 may be the next drug target for the treatment of CRC and other related cardiovascular diseases.

## 1. Introduction

Malignant tumors and cardiovascular and cerebrovascular diseases are the main diseases endangering human health [1, 2]. Due to the progress of modern tumor treatment methods, the 5-year survival rate of tumor patients has been significantly improved [3]. However, the impact of chemotherapy-related cardiotoxicity (CRC) on patients is also increasingly prominent [4]. Most patients have histological changes before the decline in cardiac function, accompanied by irreversible injury, and miss the best time window of treatment [5]. In the process of CRC, many chemotherapeutic drugs cause disordered myocardial energy and ion metabolism in the metabolic process of cardiomyocytes, resulting in extensive changes in myocardial structure and finally leading to cardiac dysfunction [5, 6]. Our previous studies have shown that abnormal cardiomyocyte apoptosis may be the ultimate executor of cardiac injury, but what and how apoptosis is caused have not been fully clarified [7, 8]. Therefore, in-depth study of the pathogenesis of CRC and early prevention and treatment of CRC have extremely important theoretical and clinical significance.

As the main pathway of intracellular protein degradation, the ubiquitin proteasome system (UPS) regulates almost all life cycle activities in organisms, including apoptosis, inflammation, transcriptional regulation, signal transduction, and the cell cycle [8, 9]. In particular, when the myocardium is damaged, the myocardial UPS system is significantly activated to regulate the generation and degradation of a large number of signal proteins, which directly affects energy metabolism, the inflammatory response, apoptotic autophagy, myocardial interstitial fibrosis, and so on [9–11]. Therefore, regulating the production and degradation of key proteins in CRC through UPS may be an important entry point to solve its occurrence and development.

As the most critical component of the UPS, ubiquitin ligase E3 is responsible for the connection between protein substrates and the UPS pathway, which determines the specific degradation of protein substrates by the UPS [12, 13]. E3, by determining the timeliness and specificity of ubiquitination, can target, recognize, and promote the degradation of substrate proteins; affect the posttranscriptional regulation of genes; and regulate myocarditis, apoptosis, fibrosis, etc. [13–15]. Therefore, the selection of the appropriate ligase E3 for intervention or specific regulation of key proteins in the occurrence and development of CRC is helpful to clarify the key molecular mechanism(s) and explore new prevention and treatment targets.

RING finger protein 10 (RNF10) is a member of the ring finger protein family (ring E3s) in ubiquitin ligase E3 [16, 17]. Its gene is located in the long arm of chromosome 12 (12q24.31), which is widely expressed in various tissues of mammals [16]. For example, the homology of the RNF10 amino acid sequence between mice and humans may be up to 90% [17, 18]. Its structure consists of two parts: a tyrosine kinase-binding domain (TKB domain) and a ring domain [19]. The ring domain is the most characteristic structure with a ring finger domain. Its function is to recruit ubiquitin ligase E2 and substrates [17, 19]. The heart develops from the cardiac plate of the embryo body, which folds into the endocardial tube. The source of the germ layer is homologous to the development of blood vessels, which is an important expression site of RNF10 [17, 20–22]. Our previous studies confirmed that RNF10 is an important regulator in the vascular remodeling model of diabetes. Overexpression of RNF10 significantly reduces carotid intima formation in diabetic rats after balloon injury and vice versa. The mechanism may be related to regulating the cell cycle, mediating the inflammatory response, and inducing cell proliferation and apoptosis [17, 21, 22]. Therefore, we speculate that RNF10 may also be involved in the regulation of cardiomyocyte structure and function and play an important role in the process of cardiomyocyte apoptosis. At the same time, it plays an important role in CRC and apoptosis. Therefore, studying the regulatory mechanism of RNF10 in CRC may have very important basic and clinical significance.

## 2. Materials and Methods

### 2.1. Materials

From Shanghai Aladdin Reagent Co., Ltd., THP was purchased (Shanghai, China). The Nanjing Jiancheng Bioengineering Institute sold a commercial lactate dehydrogenase (LDH, A020-2-2) kit. Jiangsu Meibiao Biotechnology Co., Ltd. (Jiangsu, China) sold test kits for cardiac troponin T (cTnT, MB-7278A), brain natriuretic peptide (BNP, MB-1608A), and creatine kinase MB (CK-MB, MB-6930A). Biosharp developed the CCK-8 cell viability and toxicity detection kit (BS350B) (Anhui, China). Absin (Shanghai, China) sold the antibodies RNF10 (abs127972); Proteintech (Wuhan, China) sold the antibodies activator protein-1 (AP-1, 24909-1-AP), mesenchyme homeobox 2 (Mexo2, 12449-1-AP), B cell lymphoma-2 (Bcl-2, 12789-1-AP), and Bcl-2-associated X (Bax, 9664s).

### 2.2. Animal Studies

#### 2.2.1. Animal Model and Diet

The Chongqing Medical University's First Affiliated Hospital's Animal Care Use Committee approved the studies. The researcher conducted a blind study regarding the group assignment during the animal experiment. The Chongqing Medical University Experimental Animal Center sold a total of 20 male SD rats (180–200 g, 6–8 weeks). The rat groups were as follows: normal (ND) group (rats were injected with an equal volume of normal saline through the caudal vein once a week, *n* = 10) and THP group (rats were injected with 3 mg/kg THP through the caudal vein once a week, *n* = 10). The survival of rats was recorded every day, and the food consumption and body weight were recorded twice a week.

#### 2.2.2. Electrocardiogram and Doppler Echocardiography

The lead IV ECG and Doppler echocardiography of the rats in each group were assessed at the end of the eighth week while anesthetized (inhaled isoflurane, the initial dose and maintenance dose were both 2 percent) using the BL-420F biological function measurement system (Chengdu Taimeng Science and Technique Company) and the Vivid E95 ultrasonic diagnostic apparatus (General Electric Company).

#### 2.2.3. Sample Collection and Preparation

Rats that had been fasting overnight were sacrificed by cervical dislocation (inhalation of 2% isoflurane). Blood samples from rats were collected and centrifuged at 3000 rpm for 30 min at 4°C to separate the serum.

#### 2.2.4. Biochemical Analysis

BNP, CK-MB, cTnT, and LDH levels in the serum were assayed according to the kit protocols.

#### 2.2.5. Histological Analysis and TUNEL Staining

Heart tissue was cleaned with saline solution before being preserved in 10% formalin. The heart tissue was paraffin embedded through fixation, dehydration, transparency, wax penetration, and embedding and finally sectioned into 4-5 *μ*m thick paraffin sections. Then, paraffin sections were used for hematoxylin and eosin (H&E) staining for histopathology. The TUNEL staining procedure was carried out in accordance with the TUNEL staining kit's instructions. With a Nikon Eclipse 80i microscope (Nikon, Chiyoda, Japan) magnified 200x, staining images were viewed.

#### 2.2.6. Immunohistochemistry

The prepared heart tissue was dewaxed for antigen repair, and then, the heart tissue was sealed in the sealing solution at 37°C for 30 min. After cleaning, the tissue was incubated in the primary antibody diluent of RNF10 at 4°C overnight. The next day, the heart tissue was incubated with a secondary antibody at 37°C for 30 minutes. After cleaning, DAB color development, redyeing, dehydration, and sealing were carried out. Staining images were observed with a Nikon Eclipse 80i microscope (Nikon, Chiyoda, Japan) at 200x magnification.

### 2.3. Cell Experiments

#### 2.3.1. Small Interfering RNA Processing

In this study, the RNF10 gene was silenced by small interfering RNA (siRNA). The specific siRNAs of RNF10 and nontargeted control siRNA were purchased from Tsingke Biotechnology Co., Ltd. (Beijing, China). H9C2 cardiomyocytes were transduced with Lipofectamine™ 3000 reagent (Thermo Fisher Scientific Inc., Waltham, MA, USA) according to the manufacturer's instructions. In short, H9C2 cardiomyocytes at a density of 1 × 10^6^ cells/well were inoculated into 6-well plates overnight. When H9C2 cardiomyocytes grew to approximately 50% confluence, Lipofectamine™ 3000 and an Opti-MEM medium mixture, small interfering RNA, and Opti-MEM medium were prepared. Then, the two mixtures were incubated at room temperature for 15 min and added to the cells. H9C2 cardiomyocytes were transfected at 37°C for 24 hours and treated with THP (5 *μ*M) for 24 hours.

#### 2.3.2. Construction and Transfection of Lentivirus

Lentivirus particles carrying RNF10 (Lv-RNF10) and empty vector (Lv-CON) were constructed by Beijing Qingke Biotechnology Co., Ltd., and the lentivirus vector was transferred into H9C2 cells in the presence of 15 *μ*g/mL polybrene with a complex number of infections (MOI) of 10. The growth fluid was changed 24 hours after infection. After 72 h, H9C2 cardiomyocytes were screened with 2.0 *μ*g/mL purine and cultured in a humidified incubator (95% air, 5% carbon dioxide, 37°C).

#### 2.3.3. H9C2 Cardiomyocyte Grouping and Treatment

In short, H9C2 cardiomyocytes were divided into 10 groups: CON group (control), THP group (5 *μ*M THP, 24 h), Si-CON group (nontargeted control siRNA, 24 h), Si-THP group (nontargeted control siRNA+5 *μ*M THP, 24 h), Si-RNF10 group (siRNAs of RNF10, 24 h), Si-RNF10-THP group (siRNAs of RNF10+5 *μ*M THP, 24 h), Lv-CON group (lentivirus particle empty vector, 24 h), Lv-THP group (lentivirus particle empty vector+5 *μ*M THP, 24 h), Lv-RNF10 group (lentivirus particles carrying RNF10, 24 h), and Lv-RNF10-THP group (lentivirus particles carrying RNF10+5 *μ*M THP, 24 h).

#### 2.3.4. A CCK-8 Kit Was Used to Determine the Cell Viability

According to the instructions of the CCK-8 test kit, the cell viability of cardiomyocytes in each group was detected. The cell viability of CON was defined as 100%.

#### 2.3.5. Apoptosis Was Detected by Flow Cytometry

After collecting the cells from each group, Annexin V-FITC binding solution was added to resuspend the cells, and the cells were incubated at room temperature in the dark for 10 min. After centrifugation, Annexin V-FITC binding solution was added again to resuspend the cells, propidium iodide staining solution was added to mix well, and flow cytometry was carried out in the dark at 4°C. Annexin V-FITC is green fluorescence, and DAPI is red fluorescence.

#### 2.3.6. Immunofluorescence

According to the abovementioned grouping, cell creep was made, and the cell creep was fixed with 4% paraformaldehyde for 15 min. Then, the sections were soaked in 0.5% Triton X-100 at room temperature for 20 min and then sealed at room temperature in goat serum for 30 min. Then, RNF10 primary antibody was added and incubated overnight at 4°C. The next day, the cells were incubated with the fluorescent secondary antibody at room temperature for 1 h and then with DAPI staining solution for 5 min. Finally, the samples were sealed with antifluorescent quenching agent, and the images were observed and collected under a fluorescence microscope. Staining images were observed with a Nikon Eclipse 80i microscope (Nikon, Chiyoda, Japan) at 200x magnification.

### 2.4. Real-Time qPCR

For real-time qPCR, total RNA was extracted from frozen pulverized rat hearts and H9C2 cardiomyocytes using TRIzol (Invitrogen) and then transcribed by a two-step method with a SuperScript™ First-Strand Synthesis System. The PCR products were quantified with SYBR Green PCR Master Mix (Applied Biosystems), and the results were normalized to *β*-actin gene expression. The primer sequences were as follows: *β*-actin-F: CTCTTCCAGCCTTCCTTCCT; *β*-actin-R: AGCACTGTGTTGGCGTACAG; RNF10-F: ATTTTAGCAACCAGTCCCGTCG; and RNF10-R: CCTCATCCCGTCTTCCACCAT.

### 2.5. Western Blotting

Cardiac tissue and H9C2 cardiomyocytes were lysed in radioimmunoprecipitation lysis buffer containing 1% protease inhibitor to obtain a pure protein solution. Then, a BCA kit was used to determine the protein concentration. In total, approximately 40 *μ*g of heart tissue lysate or 20 *μ*g of cell lysate was used for 12% sodium dodecyl sulfate–polyacrylamide gel electrophoresis, and then, the proteins were transferred to PVDF membranes. After blocking with rapid protein-blocking solution, the following primary antibodies were added and incubated: RNF10 (1 : 1000), AP-1 (1 : 1000), Mexo2 (1 : 1000), cleaved caspase-3 (1 : 1000), Bax (1 : 1000), Bcl-2 (1 : 1000), and GAPDH (1 : 5000). Subsequently, membranes were added and incubated in HRP-conjugated goat anti-rabbit IgG (H+L) secondary antibodies (1 : 10,000; Thermo Fisher Scientific, Inc.; 31460). BeyoECL Plus (Beyotime Institute of Biotechnology) and Image Lab software (version 5.2.1; Bio-Rad Laboratories, Inc.) were used to analyze protein expression. The specific protein expression levels were normalized to GAPDH.

### 2.6. Statistical Analysis

Data are expressed as the mean ± standard error of the mean. The normal distribution and homogeneity of variance of the data were detected using one-way or two-way ANOVA, and Tukey's multiple comparison post hoc test was used to analyze the significant differences between the groups. *P* ≤ 0.05 was considered statistically significant.

## 3. Results

### 3.1. THP Decreased the Body Weight, Food Intake, and Survival Rate of Rats

**Figure 1:**
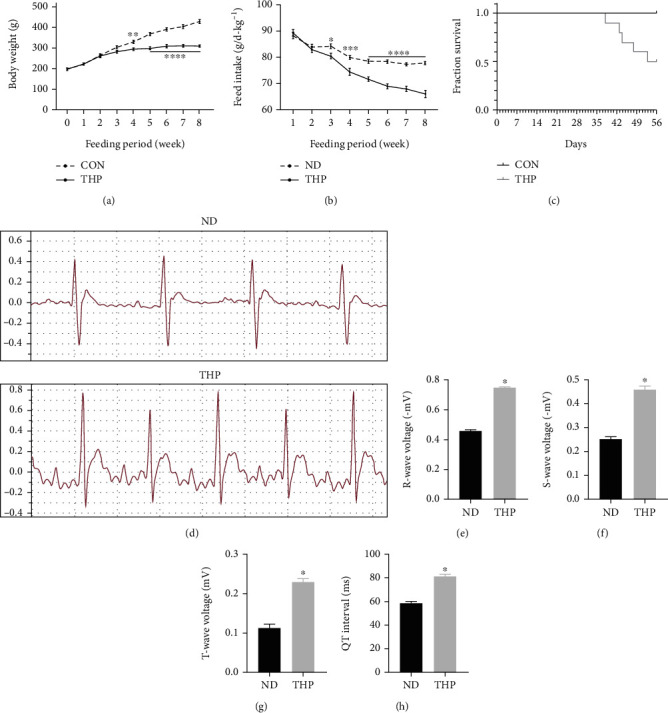
THP causes general conditions and ECG abnormalities in rats. Compared with the ND group, THP resulted in a decrease in the (a) body weight and (b) food intake of rats. In addition, one rat in the THP group died every day on the 38th, 43rd, 44th, 48th, and 53rd days, while no death occurred in the ND group (c). (d) ECG showed that compared with the ND group, rats in the THP group had elevated (e) R waves, (f) S waves, and (g) T waves and (h) prolonged QT intervals. Values are expressed as the mean ± SEM. (a, b) *n* = 10: two-way ANOVA; (c–h) *n* = 3: one-way ANOVA. ^∗^*P* < 0.05, ^∗∗^*P* < 0.01, ^∗∗∗^*P* < 0.001, and ^∗∗∗∗^*P* < 0.0001 vs. ND.

During the 8-week model establishment period, THP led to body weight loss in the rats (Figure 1(a)). The difference was significant from the 4th week, and the difference was more significant from the 5th week. Similarly, THP reduced the food intake of rats (Figure 1(b)). The difference was significant from week 3, and the difference was more significant from week 4.

In addition, normal rats were generally in good condition during the 8-week test, without obvious abnormalities and with no death. In THP-treated rats, the general state of the rats was poor, and the mortality was high, up to 50% (Figure 1(c)).

### 3.2. THP Caused ECG and Echocardiographic Changes in Rats

**Figure 2:**
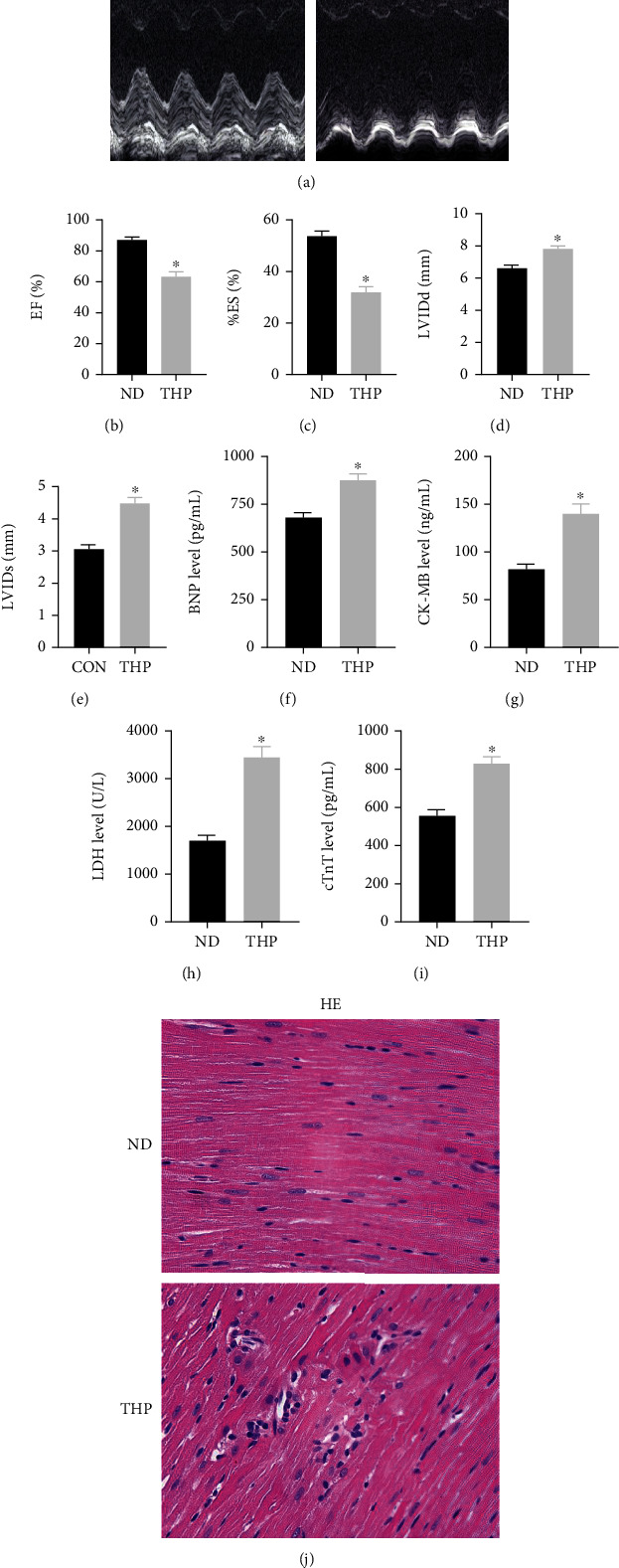
THP induced cardiac dysfunction in rats. (a) Echocardiography showed that compared with the ND group, THP caused a decrease in (b) EF and (c) FS, while (d) LVIDd and (e) LVIDs thickened. In addition, compared with the ND group, (f) BNP, (g) CK-MB, (h) cTnT, and (i) LDH in the THP group were abnormally increased. (j) HE staining showed that compared with the ND group, the morphology of heart tissue in the THP group was abnormal. Values are expressed as the mean ± SEM. One-way ANOVA (*n* = 3). ^∗^*P* < 0.05 vs. ND.

At the end of model establishment, THP caused ECG (Figure 1(d)) and echocardiography (Figure 2(a)) abnormalities in rats. The specific manifestations were the elevation of the R wave (Figure 1(e)), S wave (Figure 1(f)), and T wave (Figure 1(g)) and the prolongation of the QT interval (Figure 1(h)); EF (Figure 2(b)) and ES (Figure 2(c)) decreased, and LVIDd (Figure 2(d)) and LVIDs (Figure 2(e)) thickened.

### 3.3. THP Leads to Abnormal Biomarkers of Myocardial Injury and Cardiac Tissue Morphology in Rats

THP caused an increase in the biomarkers BNP (Figure 2(f)), CK-MB (Figure 2(g)), cTnT (Figure 2(h)), and LDH (Figure 2(i)) of myocardial injury in rats. Similarly, THP caused abnormal cardiac tissue morphology in rats (Figure 2(j)).

### 3.4. THP Caused Abnormal Expression of the RNF10 Gene and Protein in Rat Hearts

**Figure 3:**
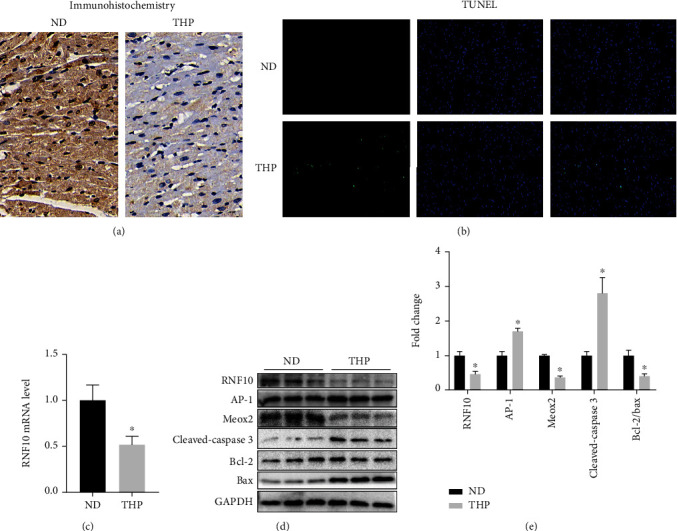
THP causes RNF10 protein and gene abnormalities and cardiomyocyte apoptosis in rat hearts. (a) Immunohistochemistry showed that THP caused a decrease in RNF10 protein expression in rat hearts compared with that in the ND group. (b) TUNEL staining showed that THP caused increased apoptosis of rat cardiomyocytes compared with the ND group. (c) PCR results showed that THP caused a decrease in RNF10 gene expression in rat hearts compared with the ND group. (d, e) WB results showed that compared with the ND group, THP caused the expression of RNF10, Meox2, and Bcl-2/Bax proteins to decrease, while the expression of AP-1 and cleaved caspase-3 proteins increased. Values are expressed as the mean ± SEM. One-way ANOVA (*n* = 3). ^∗^*P* < 0.05 vs. ND.

Immunohistochemical results showed that THP caused a decrease in RNF10 expression in the rat heart (Figure 3(a)). Similarly, the results of PCR (Figure 3(c)) and WB (Figures 3(d) and 3(e)) also showed that THP decreased the gene and protein expression of RNF10 in the rat heart. In addition, THP also resulted in increased AP-1 protein expression and decreased Meox2 protein expression (Figures 3(d) and 3(e)).

### 3.5. THP Increased Cardiomyocyte Apoptosis in Rats

THP led to an increase in cardiomyocyte apoptosis in rats, which was characterized by increased expression of cleaved caspase-3 and a decrease in the Bcl-2/Bax ratio (Figures 3(d) and 3(e)). TUNEL staining also showed that THP led to an increase in cardiomyocyte apoptosis in rats (Figure 3(b)).

### 3.6. Effect of Inhibition or Activation of RNF10 on the Activity and Apoptosis Rate of H9C2 Cardiomyocytes Induced by THP

**Figure 4:**
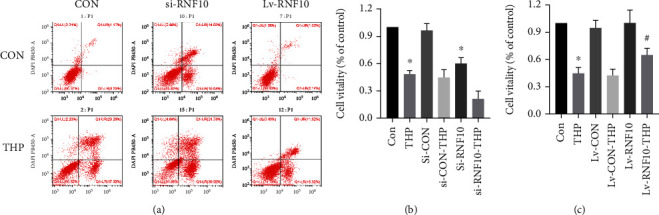
THP caused H9C2 cardiomyocyte apoptosis and decreased the survival rate. (a) The results of flow cytometry showed that compared with the CON group, THP caused an increase in the apoptosis rate, while the RNF10 silencing group also increased the apoptosis rate. Compared with the THP group, the apoptosis rate of the RNF10 overexpression group decreased significantly. The results of CCK-8 showed that compared with the CON group, THP caused a decrease in cell survival, while the RNF10 silencing group also reduced cell survival (b); compared with the THP group, the cell survival rate of the RNF10 overexpression group was significantly higher (c). Values are expressed as the mean ± SEM. One-way ANOVA (*n* = 3). ^∗^*P* < 0.05 vs. ND, ^#^*P* < 0.05 vs. THP.

Flow cytometry showed that THP caused an abnormal increase in the apoptosis rate of H9C2 cardiomyocytes, while silencing the expression of RNF10 also led to an abnormal increase in the apoptosis rate of H9C2 cardiomyocytes. Overexpression of RNF10 reversed the increase in the apoptosis rate of H9C2 cardiomyocytes caused by THP (Figure 4(a)). Similarly, both THP and silencing RNF10 expression resulted in a decline in H9C2 cardiomyocyte survival (Figure 4(b)), while overexpression of RNF10 reversed the decline in H9C2 cardiomyocyte survival caused by THP (Figure 4(c)).

### 3.7. Effect of Inhibition or Activation of RNF10 on THP-Induced RNF10 Protein Expression in H9C2 Cardiomyocytes

**Figure 5:**
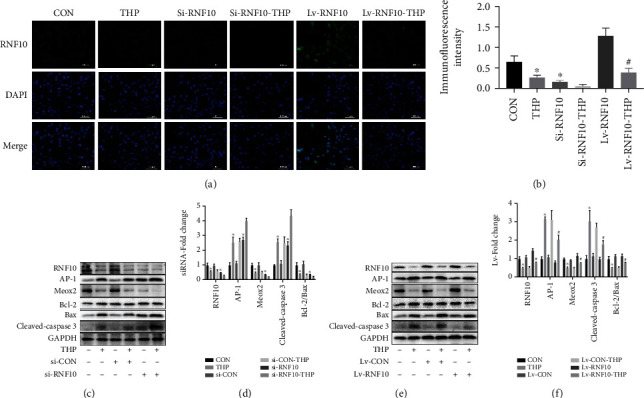
THP causes RNF10 protein abnormalities and cardiomyocyte apoptosis in H9C2 cardiomyocytes. (a, b) Immunofluorescence showed that compared with the CON group, THP caused a decrease in RNF10 expression, while RNF10 expression in the RNF10 silencing group also decreased; compared with that in the THP group, the expression of RNF10 in the RNF10 overexpression group was significantly increased. (c, d) WB results showed that compared with the CON group, THP and RNF10 silencing resulted in decreased expression of RNF10, Meox2, and Bcl-2/Bax proteins, while the expression of AP-1 and cleaved caspase-3 proteins increased; (e, f) compared with the THP group, overexpression of RNF10 resulted in increased expression of RNF10, Meox2, and Bcl-2/Bax protein, while the expression of AP-1 and cleaved caspase-3 protein decreased. Values are expressed as the mean ± SEM. One-way ANOVA (*n* = 3). ^∗^*P* < 0.05 vs. ND, ^#^*P* < 0.05 vs. THP.

The results of immunofluorescence staining showed that THP caused the fluorescence intensity of RNF10 to decrease, and the fluorescence intensity also decreased after silencing RNF10 (Figures 5(a) and 5(b)). Similarly, overexpression of RNF10 reversed the THP-induced decrease in RNF10 fluorescence intensity (Figures 5(a) and 5(b)).

### 3.8. Effects of Inhibition or Activation of RNF10 on THP-Induced RNF10 Expression and the Downstream Signal Transduction Pathway in H9C2 Cardiomyocytes

In cellular studies, RNF10 expression in cardiomyocytes after RNF10 silencing decreased, increased AP-1 protein expression and decreased Meox2 protein expression (Figures 5(c) and 5(d)). After silencing RNF10, the corresponding changes were more significant after THP treatment. After overexpression of RNF10, the corresponding changes were reversed (Figures 5(e) and 5(f)).

### 3.9. Effect of Inhibiting or Activating RNF10 on THP-Induced Apoptosis of H9C2 Cardiomyocytes

After silencing RNF10, cardiomyocytes showed the same apoptotic trend as the THP group, which was characterized by an increased expression of cleaved caspase-3 and a decrease in the Bcl-2/Bax ratio (Figures 5(c) and 5(d)). After silencing RNF10 and THP treatment, apoptosis was more significant. After overexpression of RNF10, the corresponding apoptosis trend was reversed (Figures 5(e) and 5(f)).

## 4. Discussion

CRC has become a serious public health problem in cancer treatment in China and the world, significantly increasing the cardiovascular incidence rate and death risk as well as the difficulty of cancer treatment [23]. Cardiomyocyte apoptosis caused by CRC is not only an important factor in the development of cardiac damage but also the main cause of cardiac dysfunction [24, 25]. In this process, the ubiquitin proteasome system may be an important entry point to solve the occurrence and development of CRC, and RNF10 may be a potential intervention target. Studying the specific regulatory mechanism of RNF10 will help to clarify the important role of ubiquitination in CRC and identify new therapeutic targets. In our study, we found that the general condition of SD rats induced by THP was poor, and the performance of cardiac function impairment (ECG, echocardiography, and biomarkers of myocardial injury) and the survival rate decreased, indicating that THP successfully induced chemotherapy-related cardiotoxicity in rats. In the further detection of the rat heart, it was found that THP decreased the expression of RNF10 gene and protein, regulated the expression of AP-1 and Meox2, and finally caused cardiomyocyte apoptosis. In vitro, we found that silencing or overexpressing RNF10 could regulate THP-induced cardiomyocyte apoptosis. Specifically, overexpression of RNF10 can inhibit AP-1 and activate Meox2 expression and ultimately reverse THP-induced cardiomyocyte apoptosis. In contrast, inhibition of RNF10 showed the opposite effect and aggravated cardiomyocyte apoptosis. The use of chemotherapy drugs can be traced back to the beginning of the last century, and research on CRC has become a hot spot in the last century and continues to this day. However, it is still a medical problem that has not been completely solved [26–28]. In nearly a century of research, it has been gradually clarified that oxidative stress and inflammation in CRC are the main initiating factors leading to cardiac progression, and cardiomyocyte apoptosis is the ultimate executor and final outcome of most CRCs [29–31]. Many researchers have tried to use antioxidants and anti-inflammatory drugs to fight CRC [24, 32]. In our previous studies, we found that apoptosis in CRC could be induced by ROS-induced changes in mitochondrial membrane permeability, but improving mitochondrial membrane permeability could not completely reverse apoptosis [7, 8]. Therefore, we speculate that there are other inducing factors of apoptosis in CRC.

RNF10 is an important member of the ring finger protein family of ubiquitin ligase E3 in UPS, which regulates almost all life activities in organisms. Cardiomyocytes are important sites of RNF10 expression [16, 33]. When the myocardium is damaged, the expression and transcription of RNF10 in the myocardial UPS system are affected, resulting in the production and degradation of a large number of signal proteins, which directly affect cardiac energy metabolism, the inflammatory response, oxidative stress, and apoptotic autophagy [17, 21, 22, 34]. The key is that RNF10 can target and regulate the expression of AP-1 and Meox2 [17, 20–22]. AP-1 is a transcriptional activator in cells and is a heterodimer composed of c-fos and c-Jun [35, 36]. Appropriately inhibiting the expression of AP-1 can effectively alleviate the cardiovascular toxicity caused by various factors, such as intimal hyperplasia, myocardial hypertrophy, hypertension, and myocardial fibrosis [37–39]. Homeobox proteins are a group of phylogenetically conserved transcription factors that regulate tissue growth and development, and homeobox genes are the main genes that control development and play a key role in the regulation of animal organogenesis and cell differentiation [40, 41]. There is no Meox2 expression in the early stage of heart development and only in the relatively late stage of heart development; that is, when the proliferation of cardiac cells begins to decline, Meox2 begins to be expressed [42]. Meox2 is involved in a variety of cellular processes, including cell differentiation and apoptosis [43, 44]. The expression of RNF10 enhanced Meox2-mediated activation of the p21WAF1 promoter, indicating that RNF10 is an activator of Meox2 [20, 45]. Our previous studies confirmed that the overexpression of RNF10 promotes the apoptosis of arterial intimal cells and significantly reduces the formation of carotid intima after balloon injury in diabetic rats and vice versa [17, 21, 22]. Surprisingly, in the rat model of CRC, the expression of RNF10 decreased, which seems to indicate that RNF10 plays a central, active role in CRC. In vivo, overexpression of RNF10 alleviated cardiomyocyte apoptosis, while silencing RNF10 promoted apoptosis, which was contrary to the results of balloon injury in diabetic rats. We speculate that this may be due to the difference in the role of RNF10 in different cell types. In general, RNF10 is a gene or protein that plays a positive role in life activities. Just as the human body needs normal and healthy myocardial cells without excessive hyperplasia of the endocardium, the UPS where RNF10 is located is an orderly whole and always maintains dynamic balance.

Therefore, we believe that RNF10 may be a potential intervention target in the occurrence of CRC. It is necessary to further study what role RNF10 plays in the occurrence and development of CRC, how to participate in it, and how to play the corresponding role, as well as its specific target and mode of action. Finally, we clarify the important role of ubiquitination in CRC and identify new therapeutic targets to provide an important theoretical basis for intervention targets for the prevention and treatment of clinical CRC.

## Data Availability

The data used to support the findings of this study are available from the corresponding author upon request.
